# Hf_1−x_Si_x_O_2_ Nanocomposite Coatings Prepared by Ion-Assisted Co-Evaporation Process for Low-Loss and High-LIDT Optics

**DOI:** 10.3390/ma14102606

**Published:** 2021-05-17

**Authors:** Hongfei Jiao, Xinshang Niu, Jinlong Zhang, Bin Ma, Xinbin Cheng, Zhanshan Wang

**Affiliations:** 1 Key Laboratory of Advanced Micro-Structured Materials, Ministry of Education, Shanghai 200092, China; 1810081@tongji.edu.cn (X.N.); jinlong@tongji.edu.cn (J.Z.); mabin@tongji.edu.cn (B.M.); chengxb@tongji.edu.cn (X.C.); wangzs@tongji.edu.cn (Z.W.); 2 Institute of Precision Optical Engineering, School of Physics Science and Engineering, Tongji University, Shanghai 200092, China

**Keywords:** Hf_1__−x_Si_x_O_2_ nanocomposites, ion-assisted co-evaporation process, annealing, dense amorphous structure, low optical loss, high LIDT

## Abstract

Hf_1__−x_Si_x_O_2_ nanocomposites with different SiO_2_ doping ratios were synthesized using an ion-assisted co-evaporation process to achieve dense amorphous Hf_1__−x_Si_x_O_2_ coatings with low loss and a high laser-induced damage threshold (LIDT). The results showed that the Hf_1__−x_Si_x_O_2_ nanocomposites (x ≥ 0.20) exhibited excellent comprehensive performance with a wide band gap and a dense amorphous microstructure. High-temperature annealing was carried out to ensure better stoichiometry and lower absorption. Precipitation and regrowth of HfO_2_ grains were observed from 400 °C to 600 °C during annealing of the Hf_0.80_Si_0.20_O_2_ nanocomposites, resulting in excessive surface roughness. A phenomenological model was proposed to explain the phenomenon. The Hf_1__−x_Si_x_O_2_ nanocomposites (x = 0.3 and 0.4) maintained a dense amorphous structure with low absorption after annealing. Finally, a 1064-nm Hf_0.70_Si_0.30_O_2_/SiO_2_ high-performance reflector was prepared and achieved low optical loss (15.1 ppm) and a high LIDT (67 J/cm^2^).

## 1. Introduction

Optical coatings are enabling technologies for advanced laser systems, such as laser fusion facilities [1], gravitational wave detection systems [2], and ultra-short and ultra-intense lasers [3]. Their performance is highly dependent on the properties of the low- and high-index materials that constitute the multilayer coatings. It is desirable for the coating material to have a dense amorphous microstructure with low loss [4,5,6], a wide bandgap [7], and a high LIDT [8]. The low-index material SiO_2_ that is appropriately prepared using energetic deposition meets all the above requirements [9]. However, it is difficult to obtain high-index material with excellent comprehensive properties. Ta_2_O_5_ films prepared using energetic deposition exhibit a fine and dense amorphous microstructure with low loss [10,11]. However, the bandgap of Ta_2_O_5_ is narrow, resulting in unsatisfactory laser damage resistance [11]. By contrast, HfO_2_ has a superior bandgap and LIDT [12] and is widely used in coatings for high-power lasers at facilities such as the National Ignition Facility [13] or Laser MegaJoule [14]. Nonetheless, it is still quite challenging to obtain a dense and amorphous microstructure of HfO_2_ with extremely low absorption and scattering loss.

Two approaches have been proposed to modify the HfO_2_ properties to improve its performance. In nanolamination, thin amorphous layers are added to HfO_2_ coatings to suppress crystallization during HfO_2_ growth and achieve a smooth surface with low scattering loss [15]. However, this method fails to adjust the bandgap, and the numerous interfaces in the nanolaminate reduce the LIDT, electrical property, and thermal conductivity. The fabrication of nanocomposites is another approach in which amorphous material, such as SiO_2_, is doped with HfO_2_ to modify its structural, optical, and electrical properties dramatically. Several works already demonstrated the high potential of HfO_2_-SiO_2_ nanocomposite coating applications.

Ion beam sputtering (IBS) technology has been widely used for the deposition of mixtures of HfO_2_ and SiO_2_ because it enables the formation of dense nanocomposites with high stability and repeatability. For instance, Jensen et al. [16,17,18] experimentally demonstrated that mixed HfO_2_ with SiO_2_ prepared by IBS can effectively reduce film absorption and promote LIDT for nanosecond and femtosecond pulses. Abromavicius et al. [19] studied the effects of high temperature annealing on the spectral, microstructural and LIDT properties of sputtered HfO_2_ and HfO_2_-SiO_2_ mixture-based ultraviolet (UV) mirrors, demonstrating the high potential of HfO_2_-SiO_2_ nanocomposites for the modifications of LIDT, absorption and surface topography. However, investigations of the optical loss of co-sputtered Hf_1__−x_Si_x_O_2_ nanocomposites have been rare. Moreover, the IBS process is not suitable for the preparation of large-size optical components, because the area of the target material bombarded by the ion beam is too small, resulting in poor film thickness uniformity in the preparation of large-size optical elements; additionally, the fabrication cost is relatively high.

Many studies have focused on the HfO_2_-SiO_2_ nanocomposites fabricated by electron beam evaporation (EB). For example, Jena et al. [20] performed a detailed investigation of the optical properties and laser damage threshold of HfO_2_-SiO_2_ nanocomposites. It was observed that the roughness, bandgap, and LIDT of HfO_2_ are dramatically improved by the addition of SiO_2_. Tokas et al. [6] reported on the morphological evolution and analyses of the HfO_2_-SiO_2_ nanocomposites at various mixing compositions and concluded that the HfO_2_-SiO_2_ binary composite system has great potential for ultraviolet optical coating applications that require a thin film microstructure with low light scattering. Zeng et al. [21] demonstrated the potential of the HfO_2_-Al_2_O_3_ and HfO_2_-SiO_2_ nanocomposites in improving the LIDT of dichroic laser mirrors. However, the coatings prepared by the EB process have a porous and loose microstructure that gives rise to a high scattering loss and poor environmental stability [22].

By contrast, the ion-beam assisted deposition (IAD) process may have greater potential for the fabrication of low-loss laser films, because high-energy ion bombardment will increase the packing density of the HfO_2_ films. The issue is more complicated and quite challenging for obtaining low absorption, amorphous and high-LIDT HfO_2_ prepared by IAD, because HfO_2_ films prepared by IAD always show a polycrystalline and rough microstructure [23] and high absorption [24]. The use of HfO_2_-SiO_2_ nanocomposites only solves some of these issues of HfO_2_ films prepared by IAD, and annealing is always needed to improve the performance. However, detrimental phenomena such as recrystallization may occur during annealing, leading to a loss of the desired properties [25,26].

To the best of our knowledge, no systematic studies on the HfO_2_-SiO_2_ nanocomposite coatings have been performed to date and, in particular, excellent comprehensive performance with low optical loss, a wide bandgap, and a high LIDT has not been achieved. Additionally, very few studies have focused on the optical properties of the HfO_2_-SiO_2_ mixture prepared by ion-assisted co-evaporation process and the total optical loss of the HfO_2_-SiO_2_ mixture. The interrelationship between the crystallization, microstructure, and absorption of HfO_2_-SiO_2_ nanocomposite coatings prepared by the ion-assisted co-evaporation process before and after annealing is the core issue of the present study.

In this work, a systematic study was designed to find a suitable approach to fabricate high-performance coatings characterized by low absorption, low scattering, and a high LIDT simultaneously. We systematically clarified the relationships between the SiO_2_ content and the refractive index, extinction coefficient, bandgap, and crystal phase structure and analyzed the evolution of the absorption and microstructure at different annealing temperatures and SiO_2_ doping ratios. HfO_2_-SiO_2_ nanocomposites with a high LIDT and low optical loss can be used in a wide range of applications in high-power laser systems, laser gyroscopes and gravitational wave detection. Additionally, due to their dense and amorphous film structure, they may enable the development of various promising applications such as high-power laser cavities used underwater, light detection and ranging exposed in outer space and other optical elements used in harsh environments. The experimental details are described in Section 2. Section 3 reported on the evolution of the characteristics of the mixed coatings and provides an example application of the Hf_1__−x_Si_x_O_2_ nanocomposites. A summary of our findings is presented in Section 4.

## 2. Materials and Methods

### 2.1. Sample Preparation and Annealing Methods

**Figure 1:**
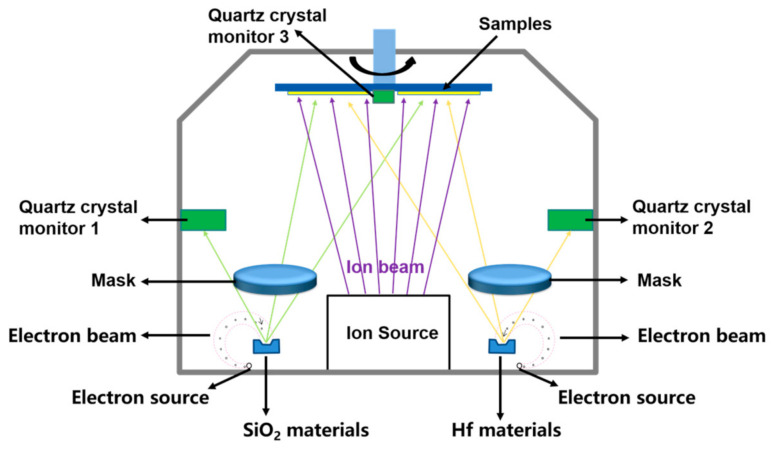
The schematic diagram of the ion-assisted co-evaporation process.

Five Hf_1__−x_Si_x_O_2_ nanocomposites with a thickness of about 300 nm were prepared with SiO_2_ doping ratios from 0% to 40%, where x represents 0, 0.1, 0.2, 0.3, and 0.4. The substrates used in this study were super-polished fused silica. The coating chamber was a modified Optorun OTFC-1300 box coater (Tokyo, Japan). The schematic diagram of the ion-assisted co-evaporation process is shown in Figure 1. Two 10-kW electron-beam guns were located at opposite sides of the chamber, and two shield plates were placed between the two guns to avoid cross-contamination. A 17-cm diameter radio frequency (RF) ion source was installed between the two guns to adjust the atomic energy of the material during the evaporation. A quartz crystal monitor was used for each gun to monitor the evaporation rate. The evaporation rate of each gun was controlled by a computer through the feedback signal from the quartz crystal monitor. A third quartz crystal monitor located in the center of the dome was used to monitor the coating’s total evaporation rate and thickness. After the modification of the coater, the two materials can be deposited simultaneous, and the Hf_1__−x_Si_x_O_2_ nanocomposite coatings were fabricated by controlling the two e-beam guns. A change in composition was achieved by changing the deposition rates of the two materials. The proportional, integral, and differential parameters of the thickness, as well as the process control systems, were judiciously optimized to suppress unwanted fluctuations in the evaporation rate. The baseline vacuum level was 3 × 10^−4^ Pa, and the substrate temperature was 200 °C. The evaporation sources included 99.95% pure Hafnium metal rods provided by Grinm Group Corporation Limited (Beijing, China) in the right crucible and 99.99% pure SiO_2_ particles provided by Feilihua Shichuang Technology Co., Ltd., Shanghai, China in the left ring crucible. We increased the current and voltage of the ion beam-assisted deposition to 1000 mA and 1100 V, respectively, to reduce the porosity and increase the packing density of the film.

XLE3648 furnace (L&L, Aston, PA, USA) was used for the annealing of the samples, and its temperature control accuracy could reach ± 3%. In the annealing process, the samples were, respectively, annealed in a quartz culture dish air for a fixed time of 24 h with the annealing temperature increased from 300 to 600 °C with a step of 100 °C. The heating rate was 5 °C/min from the room temperature to the annealing temperature and natural drop in the aerosphere was used as the cooling process.

### 2.2. Characterization

The reflectance and transmittance spectra of the samples were measured in the range 190–1300 nm at a close to normal angle of incidence by a spectrophotometer (Cary 5000, Varian, Palo Alto, CA, USA), and the refractive index, extinction coefficient, and thickness of the films were deduced from the spectra using Optichar, the optical characterization software (version 9.96) from Opitlayer (OptiLayer GmbH, Garching, Free State of Bavaria, Germany) [27]. The precision of the optical constants and film thickness was estimated at ±0.01 for the refractive index and ±0.0001 for the extinction coefficient [28]. The optical bandgap of the films was determined using the Tauc algorithm [20,29].

The crystal phase microstructure was determined by an X-ray diffractometer (XRD, Rigaku, Tokyo, Japan), and Cu Kα (λ = 0.15405 nm) and Ni were used as the target material and filter, respectively. The tube voltage and current were 40 kV and 100 mA, respectively, and the scanning speed was 2°/min at a scanning angle ranging from 20° to 80°. Atomic force microscopy (AFM, Bruker, Madison, WI, USA) measurements were performed to determine the surface topography of the Hf_1__−x_Si_x_O_2_ nanocomposites before and after annealing. The three-dimensional AFM images were obtained in the ScanAsyst mode using a Bruker Dimension Icon atomic force microscope (Bruker, Madison, WI, USA). The scan line consisted of 512 points, and the scan areas were 20 µm × 20 µm. Each sample was tested at three different surface positions to confirm the uniformity of the sample. For different positions of the same sample, the surface morphology was consistent and the variation in the surface roughness was within 0.2 nm. The typical and clearest atomic force map was chosen to describe the surface morphology and the average of the three surface roughness values was used to describe its final surface roughness. Photo-thermal technology was used to evaluate the absorption of the Hf_1__−x_Si_x_O_2_ nanocomposites before and after annealing. The wavelength of the pump beam was 1064 nm. The average absorption of the coatings was derived from four 0.5 mm × 0.5 mm areas of each sample.

## 3. Results

### 3.1. Optical Properties

**Figure 2:**
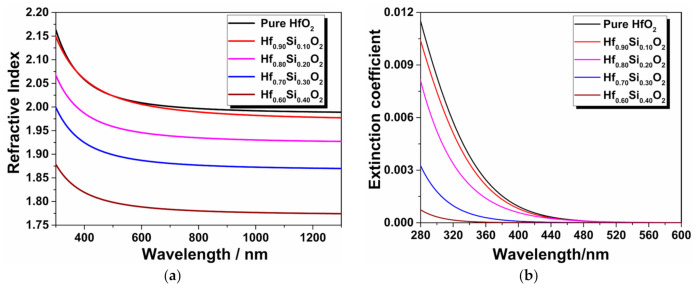
Refractive index (**a**) and extinction coefficient (**b**) dispersion curves of the Hf_1__−x_Si_x_O_2_ nanocomposite with different SiO_2_ contents.

We first describe the results of the refractive index and extinction coefficient analyses of the sets of Hf_1__−x_Si_x_O_2_ nanocomposites. The obtained refractive index and extinction coefficient dispersion curves are shown in Figure 2 indicating that the effects of doping different volume fractions of SiO_2_ on the refractive index variable and on the extinction coefficient of the ultraviolet band were significant. Both the refractive index and extinction coefficient show a normal dispersion and decrease with an increase in the SiO_2_ content since silica has a lower refractive index and is less absorptive than hafnia. However, the refractive index spectra show that the refractive index of the Hf_0.90_Si_0.10_O_2_ nanocomposites is slightly higher than that of the pure HfO_2_ film in some spectral bands. The reason is that in these composites, the local structure and microstructural evolutions play a relatively large role in the dielectric functions. On the one hand, the addition of low refractive index SiO_2_ materials will cause a decrease in the refractive index. On the other hand, the bond lengths of Hf-Hf and Hf-O are shorter for the Hf_1__−x_Si_x_O_2_ nanocomposites than the pure HfO_2_ film, resulting in a denser structure with smaller grains and a higher refractive index [20,30]. The combination of these two mechanisms led to the evolution of the refractive index of the Hf_1__−x_Si_x_O_2_ nanocomposites. This demonstrates the advantages and potential of the Hf_1__−x_Si_x_O_2_ nanocomposites in tailoring the refractive index and decreasing the extinction coefficient of the ultraviolet band. It has to be pointed out that the Hf_1__−x_Si_x_O_2_ nanocomposites has a lower refractive index compared with pure HfO_2_, which will limit their application in certain scenarios.

**Figure 3:**
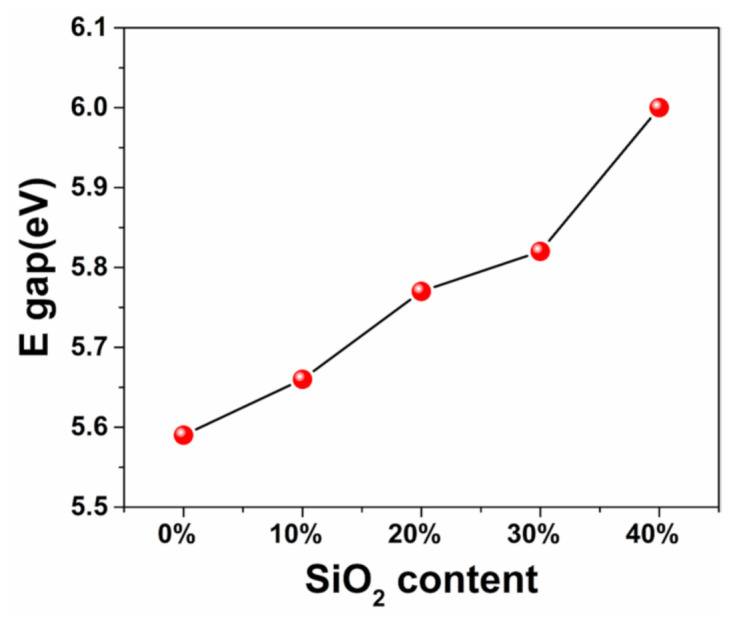
Bandgap of the Hf_1__−x_Si_x_O_2_ nanocomposite with different SiO_2_ contents.

The trend of the bandgaps and SiO_2_ content shown in Figure 3 indicate that the bandgaps increased gradually with the increase in the SiO_2_ content. The maximum bandgap of the Hf_0.60_Si_0.40_O_2_ nanocomposite was 6.01ev. It is commonly recognized that if the bound electrons in the forbidden band absorb enough photon energy, they will be excited to the conduction band, leading to multiphoton ionization, which is the prerequisite for laser damage. The wider the band gap between the forbidden band and the conduction band, the more photon energy should be absorbed during this process, and the higher the LIDT [7,31]. Therefore, these results demonstrate the advantages and potential of the Hf_1__−x_Si_x_O_2_ nanocomposites in improving the bandgap and increasing the damage threshold.

### 3.2. Microstructure Properties

**Figure 4:**
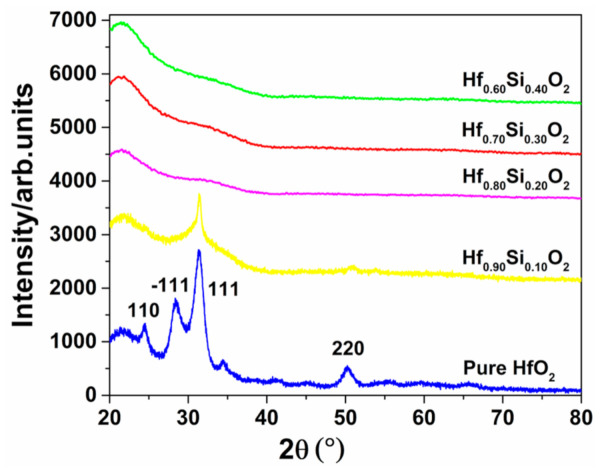
X-ray diffraction (XRD) spectra of the Hf_1−x_Si_x_O_2_ nanocomposites with different SiO_2_ contents.

The X-ray diffraction (XRD) measurements revealed the crystalline states of different SiO_2_ concentrations, as indicated in Figure 4. It was assumed that the HfO_2_ film changed from polycrystalline to amorphous with increasing SiO_2_ content. The as-deposited pure HfO_2_ coating and Hf_0.90_Si_0.10_O_2_ nanocomposite coatings exhibited polycrystalline characteristics, and the films contained the monoclinic phase of HfO_2_ with the preferred (111) orientation [32]. Moreover, the diffraction peaks in the XRD patterns of Hf_0.90_Si_0.10_O_2_ were slightly less intense than those of the as-deposited pure HfO_2_ coating. With the further increase in the SiO_2_ content, no XRD peak was found in the XRD patterns of the as-deposited Hf_0.80_Si_0.20_O_2_, Hf_0.70_Si_0.30_O_2_, and Hf_0.60_Si_0.40_O_2_ nanocomposites. Broad and amorphous-like patterns without visible peaks indicated the amorphous nature of the thin films. The XRD results revealed that the material composition of the prepared coatings strongly affected the crystal growth of the coating. The higher the SiO_2_ concentration in the Hf_1−x_Si_x_O_2_ nanocomposites, the higher was the resistance to crystallization [22]. The inhibition of crystallization was attributed to the fact that SiO_2_-doped HfO_2_ films produce many nucleation sites, preventing the growth of the crystalline phase [33,34,35]. Another reason is that Hf and Si have different ionic radii, inhibiting the formation of a long-range order structure [36].

**Figure 5:**
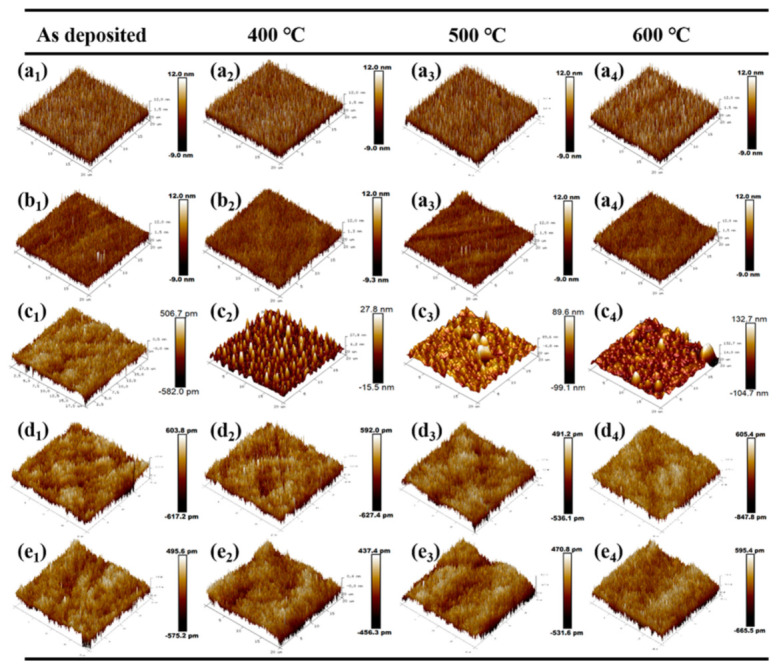
Surface morphologies of the Hf_1−x_SixO_2_ nanocomposites annealed at different temperature. (**a_1_**–**a_4_**) Pure HfO_2_ coatings, (**b_1_**–**b_4_**) Hf_0.90_Si_0.10_O_2_ nanocomposites, (**c_1_**–**c_4_**) Hf_0.80_Si_0.20_O_2_ nanocomposites, (**d_1_**–**d_4_**) Hf_0.70_Si_0.30_O_2_ nanocomposites; (**e_1_**–**e_4_**) Hf_0.60_Si_0.40_O_2_ nanocomposites.

The dependence of crystallization on the concentration of SiO_2_ in the as-deposited Hf_1−x_SixO_2_ coating has been presented as above. A SiO_2_ concentration of 20% or higher was sufficient to achieve a dense amorphous microstructure. High-temperature annealing was carried out to ensure better stoichiometry and lower absorption. In the following subsection, we discuss the evolution of the microstructure and surface roughness of the nanocomposites with different SiO_2_ contents and annealed at different temperatures. Several interesting trends were observed. The AFM maps of the Hf_1−x_Si_x_O_2_ nanocomposites before and after annealing are shown in Figure 5. It is worth mentioning that the surface morphology of all samples at the annealing temperature of 300 °C was almost the same as that of the unannealed samples; thus, we only show typical AFM diagrams of the as-deposited samples and those annealed at 400 to 600 °C. The root mean square (RMS) roughness values of the films determined from the topographic images are listed in Table 1.

We first discussed the evolution of the microstructure and surface roughness of the as deposited nanocomposites with different SiO_2_ contents. In the AFM map, we observed in Figure 5a_1_ that the as-deposited pure HfO_2_ coatings exhibited a polycrystalline structure composed of small spiky grains. These visible grains enhanced the roughness of the surface; therefore, the as-deposited pure HfO_2_ coatings had a high surface roughness of 3.92 nm. A further increase in the SiO_2_ amount inhibited the crystallization and resulted in a decay of the particles, as shown in Figure 5b_1_. As a result, the as-deposited Hf_0.90_Si_0.10_O_2_ nanocomposites had a slightly lower roughness value (2.63 nm) than the pure HfO_2_. Moreover, the surface of the amorphous Hf_0.80_Si_0.20_O_2_, Hf_0.70_Si_0.30_O_2_, and Hf_0.60_Si_0.40_O_2_ nanocomposites shown in Figure 5c_1_–e_1_, respectively, did not consist of visible particles and grains. Thus, they had a surface roughness of less than 0.2 nm and were smoother than the other as-deposited thin films, indicating the lowest scattering loss. This result is consistent with the XRD evolution.

**Figure 6:**
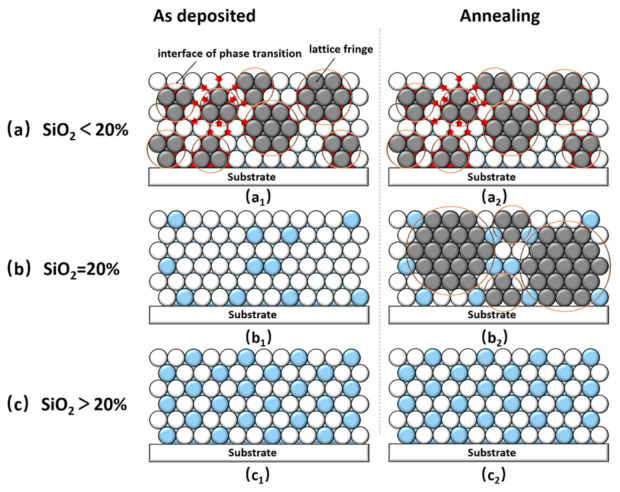
The phenomenological model describing the evolution of the crystalline states and microstructure of the nanocomposites during gradual annealing post-processing. As-deposited Hf_1−x_Si_x_O_2_ nanocomposites: x < 20% (**a_1_**), x = 20% (**b_1_**) and x > 20% (**c_1_**); Hf_1−x_Si_x_O_2_ nanocomposites after annealing: x < 20% (**a_2_**), x = 20% (**b_2_**) and x > 20% (**c_2_**).

We then investigated the evolution of the microstructure and surface roughness of the nanocomposites annealed at different temperatures. After high-temperature annealing, the microstructure of the Hf_1−x_Si_x_O_2_ nanocomposites with different SiO_2_ concentrations exhibited different evolution patterns. High-temperature annealing didn’t change the surface morphology and roughness of the pure HfO_2_ and Hf_0.90_Si_0.10_O_2_ nanocomposites, as shown in Figure 5a_1_–a_4_ and b_1_–b_4_. By contrast, precipitation and regrowth of the HfO_2_ grains were observed in Hf_0.80_Si_0.20_O_2_ nanocomposites annealed at 400 to 600 °C (Figure 5c_2_–c_4_). The aggregation of smaller grains into larger grains resulted in the uniform distribution of the nano-clusters in the whole field of view of these samples, significantly increasing the surface roughness. When the annealing temperature reached 400 °C, the RMS roughness increased abruptly to 6.12 nm, which was one order higher than that of the other samples. When the annealing temperature reached 500 and 600 °C, the RMS roughness of the nanocomposite reached about 19.1 nm and 25.7nm, exceeding the usable range of normal optical coatings. As the SiO_2_ content increased to 30% and 40%, the Hf_0.70_Si_0.30_O_2_ and Hf_0.60_Si_0.40_O_2_ nanocomposites maintained a smooth surface morphology with a minimum roughness of less than 0.2 nm, even after 600 °C annealing, as shown in Figure 5d_1_–d_4_ and e_1_–e_4_. Three states were observed in the evolution of the roughness and surface topography before and after annealing: a stable crystalline state, a transition state, and a stable amorphous state. A phenomenological model is proposed to describe the formation of the three different states (Figure 6).

In the first stage, the nanocomposite is in a stable crystalline state when the SiO_2_ concentration of the Hf_1−x_Si_x_O_2_ coatings is less than 20% (Figure 6a_1_). The deposition of the Hf molecules was completely unordered in the initial period (represented by the white spheres). The SiO_2_ molecules (represented by the blue areas) are evenly dispersed between the hafnium oxide molecules. A certain number of SiO_2_ molecules act as barriers to the diffusion of the Hf atoms and delay the nucleation and growth of HfO_2_ crystallites [37]. The AFM maps (Figure 5) and XRD (Figure 4) results also indicate that the doping of SiO_2_ suppressed the crystallization of HfO_2_, but crystallization was the dominating trend. Due to the random collisions between the atoms, short-range ordered atom clusters appeared in the coatings (represented by the dark spheres). The coatings crystallized at the base of the atom clusters, and some small fine grains formed in the as-deposited nanocomposite coating. Subsequently, after high-temperature annealing, the grain size remained almost unchanged, as shown in Figure 6a_2_. One possible reason for this phenomenon may be that the ordered and stable polycrystalline structure with higher stored energy is not conducive to the recrystallization of the nanocomposite [38]. Thus, the energy of annealing is mainly used to transfer the surface atoms to existing voids and defects, resulting in reduced surface roughness [39], as shown in Table 1. This may be the main reason why the roughness of the pure HfO_2_ and Hf_0.90_Si_0.10_O_2_ nanocomposites was slightly lower than that of the un-annealed samples at the partial annealing temperature. Another possible explanation is that the doped SiO_2_ may have induced a silicon-rich liquid phase, which acted as a segregation layer at the grain boundaries and grain junctions (represented by the red areas) during annealing. The motion of a moving grain boundary, which is dragged by a segregated solute layer and exhibits lowered mobility, inhibits the recrystallization of HfO_2_ at the depositing and annealing temperature [40], as shown in Figure 6a_2_.

Regarding the second stage, the nanocomposite is in a transition state from crystalline to amorphous when the SiO_2_ concentration of the Hf_1−x_Si_x_O_2_ coatings is around 20%, which is a critical state and represents a unique stage in the evolution. As shown in Figure 6b_1_, more and more SiO_2_ aggregates occupying an increasing number of nucleation sites were observed (represented by the blue spheres). Additionally, the number of SiO_2_ molecules surrounding the Hf atoms increased, hindering the diffusion of Hf significantly and ultimately delaying the nucleation and growth of the HfO_2_ crystallites. No silicon-rich liquid was formed during this process. The 20% SiO_2_ doping amount was sufficient to completely suppress the crystallization of the as-deposited nanocomposite, resulting in a smooth amorphous structure. However, abnormal crystallization occurred with an increase in the annealing temperature above 400 °C, as shown in Figure 6b_2_. Small grains were aggregated into large grains because the transition of HfO_2_ from an amorphous state to the crystalline state usually occurs at approximately 400 °C during annealing [25]. Thus, the amorphous as-deposited Hf_1−x_Si_x_O_2_ film did not crystallize when annealing occurred below 400 °C. The most likely reasons for this abnormal evolution pattern are as follows. The amorphous film is in a state of high free energy, which can be lowered by crystallizing into a metastable phase assembly, in which structurally complex phases require extensive atomic rearrangement [41]. Subsequently, annealing at high temperatures provided energy to the Hf atoms, enhancing the diffusion and mobility of the Hf atoms. Amorphous films in the metastable state start to crystallize without being inhibited by a segregation layer. The two mechanisms, i.e., the inhibition of crystallization by doping and the promotion of crystallization during annealing, compete with each other and play a dominant role in the transition state, thereby affecting the balance between preferential growth and random growth. Thus, there is a phase transition from amorphous to polycrystalline, and abnormal grain growth occurs at higher temperatures. Since the clusters are randomly distributed and gradually increase in size, the bulk density of the atoms increases, resulting in larger film stress. The mismatch between the stress of the film and the substrates leads to discontinuous surface topography with several gaps, as shown in the AFM images in Figure 5c_3_,c_4_.

In the third stage, the nanocomposite is in a stable amorphous state when the SiO_2_ concentration of the Hf_1−x_Si_x_O_2_ coatings is more than 20%. As shown in Figure 6c, a sufficient amount of doped SiO_2_ completely inhibits crystallization by occupying numerous nucleation sites. This occurrence plays a leading role in thermal annealing, and the energy of annealing is not sufficient to cross a potential barrier to induce crystallization. Therefore, all the as-deposited and annealed Hf_0.70_Si_0.30_O_2_ and Hf_0.60_Si_0.40_O_2_ nanocomposites maintained an amorphous structure with a smooth surface.

### 3.3. Absorption at 1064 nm

Table 2 presents the weak absorption at 1064 nm of all Hf_1−x_Si_x_O_2_ nanocomposites with different SiO_2_ contents before and after annealing. As shown in Table 2, the pure HfO_2_ film had a maximum absorption of 131 ppm. SiO_2_ doping reduced the absorption of the film substantially, and as the content of SiO_2_ increased, the absorption gradually decreased. The evolution of the absorption at 1064 nm was almost the same as the evolution of the ultraviolet (UV) extinction coefficient, as discussed in the previous section. The Hf_0.60_Si_0.40_O_2_ nanocomposite had the lowest absorption (43 ppm). After high-temperature annealing, the absorption decreased significantly with an increase in the annealing temperature for the different Hf_1−x_Si_x_O_2_ nanocomposites. When the annealing temperature reached 600 °C, all nanocomposite Hf_1−x_Si_x_O_2_ coatings, including the pure HfO_2_ coating, exhibited relatively low absorption at 1064 nm, indicating that the absorption of the Hf_1−x_Si_x_O_2_ coatings was generally reduced at high-temperature annealing. However, the Hf_0.80_Si_0.20_O_2_ coating with the 20% SiO_2_ concentration had the highest absorption (18.5 ppm) after annealing at 600 °C, which is contrary to the common belief that high-temperature annealing enhances the oxidation of HfO_2_ films and reduces the absorption loss.

**Figure 7:**
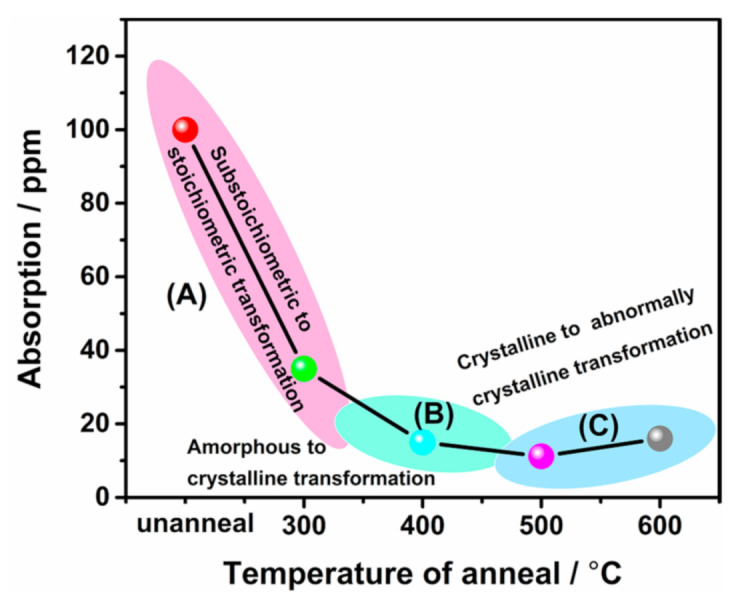
Weak absorptions of the as-deposited and annealed Hf_0.80_Si_0.20_O_2_ films at different temperatures. (**A**–**C**) represent the evolution of the films in the annealing process.

Since the weak absorption was affected by the combination of defect absorption and the microstructure of the films [42], the combination of substoichiometric defect and structural defect dominant description was used to illustrate the evolution of the weak absorption, as is shown in Figure 7. At annealing below 400 °C, the amorphous as-deposited Hf_1−x_Si_x_O_2_ film did not crystallize. The main role of annealing at this stage is to enhance the oxidation degree to achieve better stoichiometry. Thus, it is concluded that the transformation from a substoichiometric to a stoichiometric process in A tends to reduce the optical absorption considerably. This explanation applies to all Hf_1−x_Si_x_O_2_ films mentioned above, as shown in Table 1. When the annealing temperature reached around 400 °C, the structure of the Hf_0.80_Si_0.20_O_2_ nanocomposite began to change from amorphous to crystalline. Although the appearance of grain boundaries during crystal growth resulted in reduced thermal diffusion, the transformation from a substoichiometric to a stoichiometric process was not complete. Therefore, the weak absorption of the Hf_0.80_Si_0.20_O_2_ nanocomposite continued to decrease after 400 °C annealing in process B. By contrast, abnormal crystallization occurred as the annealing temperature reached 500 °C and 600 °C. Large atom clusters reduced the oxidization degree; thus, not all Hf^4+^ ions could bond with O^2−^ ions. Moreover, numerous grain boundaries also hindered thermal diffusion. The annealed Hf_0.80_Si_0.20_O_2_ nanocomposite exhibited high absorption in process C.

### 3.4. Application of the Hf_1−x_Si_x_O_2_ Nanocomposites

**Figure 8:**
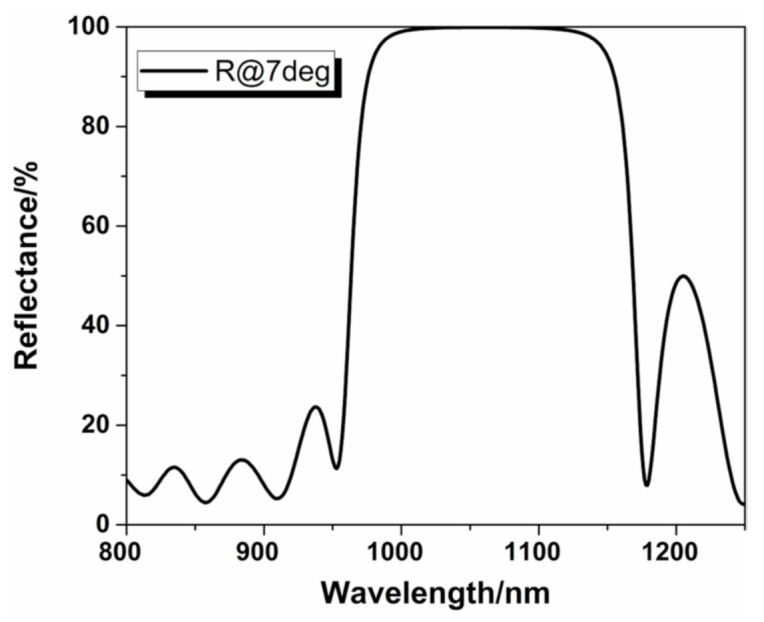
The experimental reflectance spectrum of the 1064-nm Hf_0.70_Si_0.30_O_2_/SiO_2_high reflective coating.

We have clarified the evolution of the dispersion and bandgap at different silicon oxide doping ratios and determined the evolution of the structure and absorption of the Hf_1−x_Si_x_O_2_ nanocomposite coatings during the growth and post-annealing stages. It was found that the SiO_2_ concentration significantly affected the structure evolution. When the SiO_2_ concentration exceeded 30%, after post-annealing, the Hf_1−x_Si_x_O_2_ nanocomposite fabricated by IAD had an excellent comprehensive performance with a dense amorphous structure, low absorption, low roughness, and a wide bandgap. In addition, the Hf_0.70_Si_0.30_O_2_ nanocomposite may have better potential applicability because of its higher refractive index. To demonstrate the practicability of these techniques, 1064-nm high reflective coatings consisting of SiO_2_ and Hf_0.70_Si_0.30_O_2_ were prepared by the IAD co-evaporation method and annealed at 600 °C. The film structure was sub/(LH)^17 2L/air, where H and L denote high (Hf_0.70_Si_0.30_O_2_) and low (SiO_2_) index coating materials with quarter-wave (QW) optical thicknesses at 1064 nm. The reflectance spectrum measured by a spectrophotometer is shown in Figure 8. It was observed that the reflectance of the fabricated Hf_0.70_Si_0.30_O_2_/SiO_2_ high reflective (HR) coating at 1064 nm was higher than 99.9%. Additionally, even though the Hf_0.70_Si_0.30_O_2_ nanocomposite had a lower refractive index compared to pure HfO_2_, resulting in a narrower reflection band, the HR coatings still had a sufficiently wide reflection bandwidth to satisfy practical requirements.

**Figure 9:**
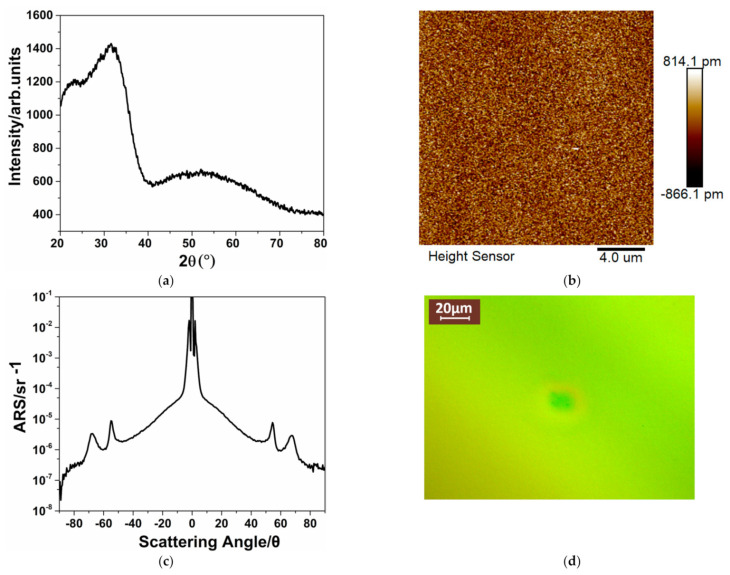
X-ray diffraction (XRD) spectra (**a**), atomic force microscopy (AFM) map (**b**), ARS (**c**), and typical damage morphology (**d**) of the 1064-nm high reflective coatings consisting of SiO_2_ and Hf_0.70_Si_0.30_O_2_ after 600 °C annealing.

An Nd:YAG laser operating at 1064 nm with the TEM00 mode was used to measure the LIDT of the final fabricated 1064 nm high reflective coatings. The Q-switched pulse width was 10 ns and the beam diameter was 1 mm in the 1/e^2^ of the laser energy. The laser damage tests were performed in 1-on-1 mode [43]. Damage was determined by a Nomarski microscope with 200× magnification. The scatterometer (ALBATROSS-TT, Hong Kong, China) was used to measure the angular resolved scatter values (ARS) of the 1064 nm high reflective coatings. The XRD spectra, AFM map, ARS, and typical damage morphology are shown in Figure 9. The absorption loss, surface roughness, scattering loss, and damage threshold are listed in Table 3.

As depicted in Figure 9a, there were no sharp diffraction peaks in the XRD spectra of the annealed sample, indicating that an amorphous microstructure was maintained during annealing. The surface morphologies illustrated in Figure 9b also showed that the annealed HR coating had a smooth surface with a roughness of 0.225 nm, resulting in a low scattering loss of 13.3 ppm (Figure 9c). Moreover, high temperature annealing effectively reduced its absorption to the level of 1.8ppm which is comparable to the fused silica substrate, as given in Table 3. The results of absorption loss and scattering loss indicated that the total loss of the HR coating at 1064 nm is as low as 15.1 ppm. The LIDT of the annealed HR coating was 67 J/cm^2^ (Table 3), which was at the same level as the pure HfO_2_/SiO_2_ HR coating prepared by EB process [17,44], and the sample exhibited “plasma induced ablation”-type representative damage morphology in the defect free region rather than the nodules induced the catastrophic damage, which reflected the rather high intrinsic damage threshold, corresponding to the low absorption and wide bandgap of the Hf_0.70_Si_0.30_O_2_ nanocomposite.

The results demonstrate the excellent comprehensive performance of the nanocomposites with low optical loss, a wide bandgap, a high LIDT and a dense amorphous structure, compared to the HfO_2_ film prepared by IBS, EB and IAD [22,23,24,44]. The material can be successfully prepared using the IAD co-evaporation method and has excellent optical and mechanical properties suitable for laser damage coatings and advanced coating applications.

## 4. Conclusions

In summary, the characteristics of Hf_1−x_Si_x_O_2_ nanocomposites with different SiO_2_ doping ratios synthesized using an ion-assisted co-evaporation process were systematically investigated, including variation trends of refractive index, extinction coefficient, bandgap, microstructure and absorption loss. The main results can be summarized as follows:

(1) For the optical properties, the refractive index and absorption coefficient were found to decrease with increasing silica content from 0% to 40%, except for the anomalous behavior of the compositions with 10% silica in some spectral bands, while the optical bandgap increases monotonically with increasing silica content. This demonstrates the advantages and potential of the Hf_1−x_Si_x_O_2_ nanocomposites in tailoring the refractive index and increasing LIDT.
(2) We analyzed the relationships between the SiO_2_ content and the microstructure at different annealing temperatures and found that the Hf_1−x_Si_x_O_2_ nanocomposites changed from polycrystalline to amorphous with increasing SiO_2_ content. A SiO_2_ concentration of 20% or higher was sufficient to achieve a dense amorphous microstructure. The microstructure of the H_1−x_Si_x_O_2_ nanocomposites with different SiO_2_ concentrations exhibited different evolution patterns during annealing. We proposed a phenomenological model to explain the microstructure evolution.
(3) We explored the weak absorption at 1064 nm of all Hf_1−x_Si_x_O_2_ nanocomposites with different SiO_2_ contents before and after annealing. SiO_2_ doping and high-temperature annealing substantially reduced the absorption of the film. When the annealing temperature reached 600 °C, all Hf_1−x_Si_x_O_2_ nanocomposites exhibited relatively low absorption. However, the Hf_0.80_Si_0.20_O_2_ nanocomposites had the highest absorption of 18.5 ppm after annealing at 600 °C. We proposed a description of the substoichiometric defects and the dominant structural defects to illustrate the absorption evolution of the Hf_1−x_Si_x_O_2_ nanocomposites with different SiO_2_ contents before and after annealing.
(4) When the SiO_2_ concentration exceeded 30%, a dense amorphous structure with a relatively low roughness value and low absorption loss was maintained after thermal annealing. We prepared 1064 nm high-reflective coatings consisting of SiO_2_ and Hf_0.70_0S_0.30_O_2_ and achieved low optical loss (15.1 ppm) and a high LIDT (67 J/cm^2^).

Our findings demonstrated the great potential of the Hf_1−x_Si_x_O_2_ nanocomposites prepared by ion-assisted co-evaporation process for a considerable improvement in LIDT and optical loss. Since these nanocomposites are adopted for optical thin films for the first time in this work, their stress and mechanical loss properties need to be studied in further work. Additionally, further experimental investigations are needed to validate their damage performance and temperature rise under high-energy continuous laser irradiation.

## Figures and Tables

**Table 1:** The roughness values of the Hf_1−x_Si_x_O_2_ nanocomposites coatings annealed at different temperatures.

Annealing Temperature	Roughness (nm)				
	X = 0	X = 0.10	X = 0.20	X = 0.30	X = 0.40
As deposited	3.92	2.63	0.153	0.186	0.203
300 °C	3.68	2.54	0.173	0.201	0.218
400 °C	3.83	2.59	6.12	0.232	0.201
500 °C	3.88	2.61	19.1	0.197	0.197
600 °C	3.75	2.35	25.7	0.225	0.216

**Table 2:** The weak absorption at 1064 nm of the Hf_1−x_Si_x_O_2_ nanocomposite coatings annealing at different temperature.

Annealing Temperature	Absorption (ppm) at 1064 nm				
	X = 0	X = 0.10	X = 0.20	X = 0.30	X = 0.40
As deposited	131.0	110.4	100.0	68.0	43.0
300 °C	72.0	55.2	35.3	14.3	9.8
400 °C	31.0	20.2	15.0	12.2	7.8
500 °C	15.0	9.5	11.2	5.0	3.4
600 °C	2.5	2.0	18.5	1.5	1.5

**Table 3:** Optical properties of the 1064-nm high reflective coatings consisting of SiO_2_ and Hf_0.70_Si_0.30_O_2_ after 600 °C annealing.

Samples	Absorption/ppm	Scattering/ppm	Total Loss/ppm	RMS/nm	LIDT/J/cm^2^
Hf_0.7_0Si_0.30_O_2_−SiO_2_ HR coatings	1.8	13.3	15.1	0.225	67 ± 2

## Data Availability

The data presented in this study are available on request from the corresponding author.
